# The interaction of personal, contextual, and study characteristics and their effect on recruitment and participation of pregnant women in research: a qualitative study in Lebanon

**DOI:** 10.1186/s12874-018-0616-5

**Published:** 2018-11-29

**Authors:** Jennifer J. Ayoub, May Abiad, Michele R. Forman, MINA Collaborators, Lara Nasreddine, Nahla Hwalla, Ghina Ghazeeri, Anwar Nassar, Khalid Yunis, Saadeddine Itani, Al Anoud Al Thani, Mariam Alabdulmalik, Amira Mahmoud Hassan, Hiba Bawadi, Gladys Honein-AbouHaidar, Farah Naja

**Affiliations:** 1 Faculty of Agricultural and Food Sciences, American University of Beirut, Beirut, Lebanon; 2 Faculty of Arts and Sciences, American University of Beirut, Beirut, Lebanon; 3 Department of Nutrition Science, Purdue University, West Lafayette, IN USA; 4 Hariri School of Medicine, American University of Beirut, Beirut, Lebanon; 5 Faculty of Agricultural and Food Sciences, American University of Beirut, Beirut, Lebanon

**Keywords:** Qualitative study, Lebanon, Barriers, Facilitators, Participation, Recruitment, Focus groups, In-depth interviews

## Abstract

**Background:**

Declining participation rates are impeding health research. Little is known about factors influencing the decision to participate in low- and middle-income countries (LMIC). Therefore, this paper reports on the various individual factors and their with contextual factors in influencing participation in research among pregnant women and the recommendations to enhance their recruitment in Lebanon.

**Methods:**

This study used a qualitative research design drawing on focus groups and in-depth interviews. The Theoretical Domain Framework guided data collection and analysis. The three participant groups included: Group 1-Pregnant women (*n* = 25) attending public pre-natal events and antenatal clinics in Beirut; Group 2-Pregnant women (*n* = 6) already enrolled in the ongoing Mother and Infant Nutritional Assessment birth cohort study; Group 3-Key informants (*n* = 13) including health care workers involved in recruiting pregnant women. Conversations were audio recorded, transcribed, translated into English, and thematically analyzed.

**Results:**

Three main factors influencing participation were revealed, with each factor encompassing several sub-themes: (1) personal factors (altruism, self-confidence, personal interest in the topic, previous understanding of the nature and purpose of research, education level, and previous research experience), (2) contextual factors (societal factors, family and friends), and (3) study characteristics (burden of the study, ethical considerations, incentives, and research interpersonal skills and physician endorsement to participate). The results suggested a dynamic interaction among the identified factors, forming two intersecting axes, with a four-quadrant configuration. The y- and x-axes represented personal factors and contextual factors, respectively. Individuals positioned on the lower-left quadrant were the least likely to participate; those on the upper-right quadrant were the most likely to participate; while those on the upper-left and lower-right quadrants were indecisive. Study characteristics seemed to affect the decision of pregnant women to participate situated in any of the four quadrants. Specific recommendations to improve participation were also identified.

**Conclusions:**

Our findings suggested an interaction of personal factors, contextual factors, and study characteristics affecting subjects’ participation. This interaction integrates factors into a novel dynamic framework that could be used in future studies. The recommendations identified may help improve participation of pregnant women in health research hence enhancing the quality and generalizability of research findings in LMIC.

**Electronic supplementary material:**

The online version of this article (10.1186/s12874-018-0616-5) contains supplementary material, which is available to authorized users.

## Background

During the last couple of decades mounting evidence suggested gestation as a critical window during the lifespan whereby environmental exposures and lifestyle behaviours of pregnant women may have significant effect not only on the growth and development pattern of the offspring but also on disease risk later in life [1–4]. Accordingly, various research and governmental agencies have proposed recommendations, policies and interventions promoting healthy behaviours during pregnancy [5, 6]. Advancements in the formulation of these recommendations, policies and interventions require strong evidence for their feasibility, efficacy and cost effectiveness. Such evidence ought to be based on rigorous population-based research such as birth cohorts and randomized clinical trials conducted among pregnant women. The challenge in subjects’ recruitment is a factor commonly reported to hamper the progress as well as the quality of these research efforts. Suboptimal participation could have profound effects on internal and external validity due to the possibility of selection bias [7–10]. In addition, a lower number of study participants could also lead to an underpowered study, where clinically important effects fail to reach significance [11].

The challenge of recruiting participants, including pregnant women, in health research has been attributed in part to individual factors including: lack of trust in the health profession/research staff [12]; concerns about data sharing and confidentiality; inconvenience due to time commitment, cost or work absenteeism, lack of information about the study [13]; a perception of personal risk, and fear of side effects [14]. In addition to the aforementioned individual factors, societal and cultural aspects have been postulated to play a pivotal role in affecting subjects’ participation in research studies [15]. Recruitment of pregnant women in birth cohort studies is further challenged by the fact that participants may perceive no direct benefit to themselves [16].

As such, the factors influencing participation in research are often context specific [17]. However, while the majority of studies that explored factors influencing the decision to participate in research has been conducted in developed countries such as the United States, Canada, and Europe [14, 18], little evidence existed from low- and middle-income countries (LMIC). With some LMIC transitioning towards a more research literate environment, researchers are faced with challenges due to limitations in funding or resource allocation, scientific expertise and difficulty in subjects’ recruitment [19]. International research collaborations between developed countries and LMIC may provide support in terms of funds and expertise, but cannot overcome the challenges in subjects’ recruitment in LMIC [20]. Therefore, a context-specific examination of the factors affecting the decision to participate in research in LMIC ought to be conducted.

Further, factors influencing participation are also population specific, including pregnant women population (Butt et al. Loxton et al.). Limited available evidence suggested that pregnant women may be interested in research but could be extra cautious about participation and/or are overwhelmed with the preparations for the arrival of their child [21, 22]. Our own research is supporting the extant literature on poor participation among pregnant women. Our recent study, Mother and Infant Nutrition Assessment (MINA) birth cohort study, is based in two countries, Lebanon and Qatar. It is the first mother and child cohort in the Middle East.. The aim is to provide evidence-based nutrition and lifestyle recommendations for pregnant women in the two countries. Pregnant women in their first trimester were recruited from private clinics, primary health care centers, and hospitals as described elsewhere [23]. However, in Lebanon, between November 2015 and June 2017, only 132 of 332 (39.8%) of eligible women were recruited. Compared to other studies of pregnant women, the daily recruitment, response and retention rates in the MINA birth cohort, were significantly lower [24, 25], raising questions about the factors which may support or detract participation of pregnant women in a LMIC context in research studies thus giving rise to the need for strategies to improve recruitment.

We conducted this qualitative study to examine the various individual factors and their interaction with other contextual factors in motivating/deterring pregnant women from participating in research and to identify the subjects’ recommendations to improve participation. Unlike quantitative research, qualitative methods adopt a holistic perspective that allows better capturing the complexities of human behaviors and actions [26]. In the context of the research objective of this study, a qualitative approach is better suited to help understand subjects’ decision to participate in research in natural settings, giving due emphasis to the meanings, experiences, and views of all the participants [27]. The results of this study will provide evidence for the formulation of strategies to improve participation of women and will ultimately enhance the quality and generalizability of research findings.

## Methods

### Study design and population

This study is based on a qualitative research design drawing on focus groups and in-depth interviews. We had three participant groups: 1) Group 1- Pregnant women attending public pre-natal events and antenatal clinics in Beirut. The focus group approach was used for the pregnant women attending public pre-natal events, while the in-depth interview approach was implemented with pregnant women attending antenatal clinics; 2) Group 2- Pregnant women who were already enrolled in the MINA birth cohort study and are attending antenatal clinics in Beirut; 3) Group 3- key informants (KIs) including health care workers involved in recruiting pregnant women at MINA birth cohort study recruitment sites. For groups 2 and 3, the in-depth interview approach was used.

### Sampling, recruitment approach, and eligibility criteria

A purposive convenient sampling approach was used to recruit participants from all three groups. All pregnant women participants in the focus groups or in the in-depth group interviews were aged 19–40 years, of Lebanese nationality or Syrian/Palestinian residing in Lebanon for more than 5 years. Additionally, MINA pregnant women had to have completed at least 2 MINA visits. The KIs -obstetricians, nurses, and research assistants- who were invited to participate in this study were involved in subjects’ recruitment for the MINA birth cohort. Research assistants invited pregnant women at public pre-natal events or in the waiting room of antenatal clinics in Beirut. MINA pregnant women were invited to participate by phone call using their contact information provided to the research team. The KIs were identified and invited via email by the Principal Investigator using a brief invitation email.

### Theoretical domains framework (TDF)

Without prior research in the region to explore the factors influencing participation in research studies and any factors likely to be within the behavioral realm, we applied the Theoretical Domain Framework (TDF) to help with data collection and analysis. TDF includes constructs from 33 behavior change theories and has 12 theoretical domains that were previously used in health care services studies [28–30]. For qualitative studies, in particular, TDF has been shown to be a useful tool for the exploration of explanations for suboptimal implementation behaviors and for the identification of suitable theories to further investigate those behaviors [28]. TDF theoretical domains include: knowledge, skills, social/professional role and identity; beliefs about capabilities and consequences; motivation and goals; memory, attention and decision processes environmental context and resources; social influences, emotional and behavioral regulation; and nature of the behavior.

A topic guide, formulated from the TDF, included the following: knowledge about medical research, skills, beliefs about the ability to and consequences of participation, motivation to participate, social influences, and characteristics of the research process (see Additional file 1). For both the focus groups and in-depth interviews, a semi-structured questionnaire was developed, based on this topic guide, to initiate and orient the discussions as well as to encourage every participant to voice his/her opinion and promote interaction. Each focus group and in-depth interview lasted on average 60 min and 30 min, respectively.

### Study protocols for focus groups and interviews

#### Focus groups

Two focus groups were conducted for pregnant women recruited from public pre-natal events. Moderation following the traditional approach recommended by Liamputtong [31] was done by GHA, who is experienced in conducting focus groups [32–34], while the senior author was the non-participant observer. No prior knowledge or characteristics about the moderator or observer were shared with participants.

Two focus groups were held consecutively with time in between for de-briefing and analysis. They were conducted in a private setting at the university or at the pre-natal event to protect privacy and enhance participation. The moderator opened the discussion, introduced herself and the observer, and explained that the role of the moderator was to guide the discussion while the observer took notes and observed the group dynamic. The moderator then proceeded to circulate a form to collect sociodemographic information including age, country of birth, highest education level, employment status, residential location, and economic status (see Additional file 2).

#### In-depth interviews

The interviewers (JJA and GHA) had no prior relation with the participants. The interviewer introduced herself and re-iterated the purpose of the study and indicated participants’ could withdraw at any time. The interviewers then circulated the sociodemographic data collection form (see Additional files 2 and 3). The interviews were conducted in a private setting at the antenatal clinics. All interviews were audio-taped, and a total of 34 interviews were conducted.

##### Data analysis

Sociodemographic characteristics were analyzed using SPSS, 2.1 Software. All focus groups and in-depth interviews were transcribed and translated into English. The analysis used a thematic approach as outlined by the TDF framework that had six phases [35]. In phase 1, GHA and JJA read and re-read each transcript to familiarize themselves with the information. In phase 2, the data coding guided by the TDF framework generated an initial list of codes (Open coding). In phase 3, GHA and JJA discussed the relationships between codes and created a log of candidate themes and sub-themes, with definitions and sample narrative illustrating each theme and sub-theme. In phase 4, the list of themes was refined through discussion among ourselves and other research team members to reach themes and sub-themes and the relationship between the themes. In phase 5, we further refined the themes. In phase 6, we provided a narrative of the findings and a synthesis of all the results in a conceptual framework. We supported our findings with quotes from interviewees for each theme and sub-theme.

##### Increasing rigor

We ensured that credibility and reflexivity were observed. In terms of credibility, focus group moderator/ in-depth interviewer shared the same first language and were gender congruent to enable participants to efficiently and easily communicate with each other during the discussion. All conversations were audio recorded, transcribed verbatim, translated into English, and used as the main data repository. The decision to stop further data collection was made when saturation was reached. As for researcher credibility, JJA was trained by the senior researcher on how to conduct interviews. In terms of reflexivity, to avoid any undue influence, the interviewer (GHA and JJA) of the in-depth interviews had no prior relationship with participants. For focus group, both GHA and JJA outlined their positions and personal assumptions before the first focus group. These assumptions were revisited during the data analysis phase. Further, during focus group moderation, the roles of the moderator and observer were disclosed to participants. Finally, all team members were involved in the analysis so as to avoid bias interpretation of the results.

The reporting of this study followed the COnsolidated criteria for REporting Qualitative research (COREQ) Checklist [36] (see Additional file 4).

## Results

### Sociodemographic characteristics

The data were collected between December 2016 and January 2017. In total, Group 1 had two focus groups for a total of 10 pregnant women attending public pre-natal events and 15 in-depth group interviews with pregnant women attending antenatal clinics; Group 2 had six in-depth interviews with pregnant women enrolled in the MINA birth cohort; and Group 3 had 13 KIs including obstetricians, nurses, and research assistants involved in the recruitment of women in the MINA birth cohort study.

In Table 1, the mean ages of participants in Groups 1 and 2 were 29 years and 32 years for them and their spouses, respectively. The majority were of Lebanese nationality, had a university degree or higher but did not have a health related major. Approximately 65% were employed, 68% had a university degree or higher, and 42% lived in Beirut. Their husbands were either self-employed or employed elsewhere, and over 50% had a mean monthly income under $2 K.

**Table 1:** Sociodemographic characteristics of pregnant women from groups 1 and 2 (*n* = 31)

Characteristics of participants	Groups 1 and 2 (n = 31)	
*Age of participant (years) (μ ± SD)*	28.97	3.98
*Husband’s age (years) (μ ± SD)*	32.29	4.81
Nationality (N, %)		
Lebanese	30	97
Highest Educational Level (N, %)		
University degree or higher	24	77
Specialized Health-Related Major (N, %)		
Yes	13	42
Employment (N, %)		
Working	20	65
Area of Residence (N, %)		
Beirut	13	42
Highest Educational Level of husband (N, %)		
University degree or higher	21	68
Husband’s Employment (N, %)		
Full-time or part-time employee	24	77
Self-employed	7	23
Monthly income of the family (N, %)		
600,001 – 1999,000 ($401 – $1332.9)	8	26
2000,000 – 2,999,000 ($ 1333 - $1999.9	8	26
≥ 3,000,000 (≥ $ 2000)	10	32

For Group 3, the mean age was 36.25 ± 8.51 years, with 75% females and 25% males. Their occupation included medical doctors, nurses, clinical assistants, and research assistants (data not shown).

#### Factors affecting the decision to participate

Results showed three main factors influencing participation, including (a) personal factors, (b) contextual factors, and (c) study characteristics. A few sub-themes emerged within each factors. Further examination of the results showed an interaction between the factors influencing participation, which will be discussed in section 3.

**Fig. 1:**
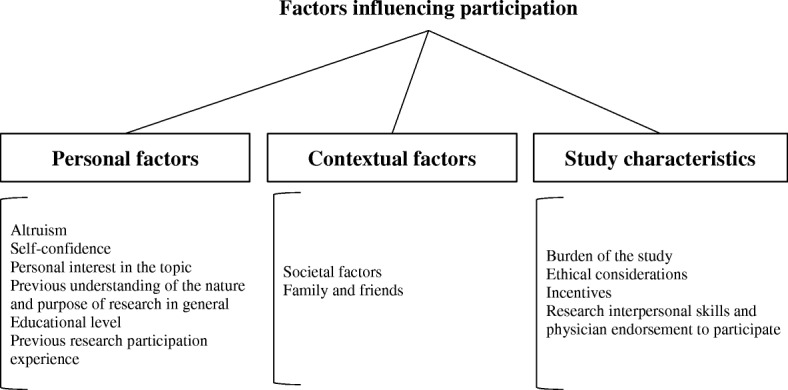
The factors influencing participation and the corresponding sub-themes

These factors and their corresponding sub-themes are represented in Fig. 1. The specific quotes corresponding to the factors influencing participation and their sub-themes are shown in Table 2.

**Table 2:** Specific quotes corresponding to the factors influencing participation and their sub-themes

Factors	Sub-theme	Quotes
Personal factors	Altruism	“I know that they take a number of people and some research on them to come up with results for a certain topic that is beneficial for the society” G2_01. “Studies might be of great benefit for the future.. These studies might help us all… that’s very encouraging in my perspective” G1_18. “The fact that my participation may be an added value to many other persons, like curing other persons or it might help others. This makes you feel you are doing something important” G1_01.
	Self-confidence	“I do, I love to be updated on new information, I read some studies online, I love to discover new results, and things that happened with doctors, for example if I am pregnant I will read about pregnancy, the foods I can eat and the foods I can’t, food sensitivities for pregnant women. I also read the medicine leaflet and ask the pharmacist about it.” G1_07. “Some people are not outspoken.. they don’t like sharing their opinions and thoughts so they simply refuse to participate in the study. At the end of the day, it is something personal… ”G2_02.
	Personal interest in the topic	“When she told me the study is about pregnancy I got excited since I’m pregnant and this topic attracts me” G2_01.
	Previous understanding of the nature and purpose of research in general	*“They don’t know what a research study is. For example, you come and tell a patient to take a certain medication and you explain to her that studies showed that it is beneficial to you. She doesn’t understand it or accept it. What is research? She doesn’t know what it means.”* G3_13. *“Here in this country and in this area of the world get scared when you tell them about a ‘study’. They directly think as if they are study subjects and that you are going to experiment on them”* *G3_05.*
	Educational level	“Yea, it’s not important to have a bachelor’s or master’s degree to be able to enroll in a study, little things matter… share personal experience and that would be beneficial.” G1_12. “Not really, in one of my recent studies I have patients of low socioeconomic status, not well educated. .. And we have educated ones that accept and also others who don’t accept .. So I don’t think there is a trend”. G3_07. “Some people are not educated, and they are not very open and would hesitate to participate in a study because they don’t know where this goes.. This is very probable. A lot of people are like this and think this way. They would consider it as a waste of time” G1_23.
	Previous research experience	“Yes, I already participated in a study before during my first pregnancy so I didn’t have any problem..” G2_06.
Contextual factors	Societal factors	
	Fostering scientific research in society	“I think in this time yes they accept since now we are in the era of science, and it’s rare now to find uneducated women or in the working environment or study environment, any educated women nowadays joined a study or conducted one in her university it is a must.” G1_14. “In my opinion, the Lebanese society is mainly open to participating in studies” G3_04. “I think the culture is very different… in Lebanon and in the Middle East in general the attitude towards research is very different than in the west… we don’t have the same level of motivation compared to the western countries” G3_12.
	Exposure to research through the media	“So, I think that there should be more emphasis and the benefit of these research. There should be awareness campaign. For example, we are doing this… for a better society, for less sickness in the country, things like that. So that people would start thinking and seeing where this is leading. This is important.” G1_01.
	Family and friends	“I might tell my husband or my friend that I participated in such a study, and some people can be understanding and encouraging, but others can dissuade you and tell you, why do you want to waste your time on such a thing?” G1_18. “I had one participant who was interested then when she told her husband on the phone that she participated in the study, 5 min after we finished the 1st visit, she came to me and told that she can’t participate anymore because her husband doesn’t want her to” G3_01. “I do believe that if family members advise her not to give out her information, she would listen to them and just not participate. So, yes, there are people who are influenced by family members.” G2_02.
Study characteristics		
	Burden of the study	
	Time commitment	“The major barrier for joining a long term study is time.. Maybe this would be the only barrier” G1_11. “When it is cross-sectional, they will think it is easy to finish for just one time. When it is a cohort, they will see it as something complicated. They do not want commitment for a long period of time” G3_11.
	Logistical inconvenience	“But leaving her baby for some time to participate in the study will be hard for her.. Leaving her baby with someone will be out of her comfort zone.” G1_11. Interviewer: “If it requires you to come and go to finish the visits”. G1_13: “This will be hard for me I can’t”.
	Type of data collected	“On the contrary. How are you supposed to do a credible study if you don’t take any sample! Questions are not enough on their own.” G2_03. “Pregnant women with hormonal rush, who might have eaten a lot the previous night, it’s really an intimate thing actually, especially that you really don’t think of what you ate until you see them written on a paper and you will be like (oh what have I done to myself)” G2_4.
	Ethical considerations	*“Telling the subjects that the information collected will remain confidential and anonymous. This also helps in encouraging people”* G1_22. *“To have everything clear to them from the beginning”* G1_24. *“Not at all, already I can skip any question I don’t want to answer, after I signed the consent I knew she can ask me whatever she wants, and she gives you the option to leave whenever you want which is relieving”* G2_01. *“When you finish with the study, tell us the results that you get”* G1_24.
		*“But I will ask them from where they are coming.. I will help them, but if they have no approval from their university to do a study, then I won’t do it.”* G1_07.
	Incentives	*“For example, if the location of the study is far, maybe providing them transportation, so yes, some would be motivated by incentives”* G1_22. *“Yes. This is a nice way to encourage people to participate in the study with the incentives”* G1_25. *“Some women get encouraged because of the incentive we give, but these people are also a minority. People who get excited because of the incentive are mainly from the Outpatient Department clinics”* G3_03. *“Yes maybe for those who need the money this would push them to participate”* G3_06.
	Researcher interpersonal skills and physician endorsement to participate	*“If the researcher is pleasant and makes you feel comfortable. If they talk to you in a nice way, if they meet them at times that suits the subjects. To be punctual on their appointments, especially if someone has a baby they can’t stay all the time and they are occupied most of the time. The personality of the researcher is very important”* G1_17.
		*“I know for a fact if the patients were asked to join by their doctor they will be more prone to say yes, since they will think that their doctor knows what he is doing and not pushing them to a random thing, which will make them more comfortable to join.”* G1_04. *“We came here [focus group] upon the request of our doctor”* G1_08.

### Personal factors

(i) Altruism:

Some pregnant women understood research to be *“something they do to discover something new”* G1_21, which is often meant to benefit the society. Many pregnant women said that what encouraged them to participate in research was knowing that their participation would *“help in the success of the study”* G1_21, and often the outcomes of studies would be of benefit for them and for their society.

(ii) Self-confidence:

Most pregnant women and a few MINA pregnant women perceived “*personality as so important*” G1_12 in the decision-making process. Our results showed that those who were self-confident, eager, and outspoken were more likely to participate in studies, while those who lacked self-confidence and were introverted were less likely to participate.

(iii) Personal interest in the topic:

Because the research topic was interesting to subjects, many participants from groups 1 and 2 were willing to participate in the study.

(iv) Previous understanding of the nature and purpose of research in general:

Although opinions varied, there was a consensus that most individuals in the Lebanese society were not familiar with understanding the nature and purpose of research. KIs indicated that the overall lack of familiarity with research was the major barrier for discussing research with potential participants. Pregnant women also agreed that most “*people are not familiar of the purpose of the research…*” G1_01:“*And yes, awareness about studies does play a role in encouraging people to participate in studies*” G2_02. Moreover, when asked to describe what research means to them, most pregnant women indicated that research is *“asking questions and com[ing] up with a percentage based on the answers”* G1_23. Very few mentioned testing an intervention, and fewer knew about cohort studies. Furthermore, a few study participants pointed to a misconception of what research is as deterrent to engagement in research.

(v) Educational level:

When asked whether they perceive the educational level to be an important factor in the decision to participate in studies, participants had conflicting opinions. Some pregnant women believed that they do not have to be highly educated in order to participate in research. One KI echoed the view that education was not a factor influencing participation. Conversely, many other pregnant women and a few MINA women pointed to formal education as an important factor to appreciate the importance of research.

(vi) Previous research experience:

Our results showed that previous participation in research encouraged participation in future studies.

### Contextual factors

(i) Societal factors:

In terms of societal factors, the study participants identified fostering scientific research in society and exposure to research through the media as factors influencing the decision to participate. When asked whether the Lebanese society is a fostering environment for research, there were opposing opinions. Many pregnant women said that our society is transitioning towards acceptance of research*,* and a few KIs echoed this opinion. Conversely, a few pregnant women indicated that *“in general the society here is very reserved about research”* G1_05, and many KIs pointed to a society that is not fully engaged in research like in the western countries. Moreover, in terms of exposure to research through the media, the study participant described that the more individuals were exposed to research results in the media -such as during show talks in television and radio-, the more likely they were to participate in research. This opinion was voiced by some participants from the three groups.

(ii) Family and friends:

Our results showed that partners acted as levers whose full support encourages individuals to be more engaged and enjoy the experience, whereas their lack of support can hamper the participants’ interest and impede their participation. KIs had firsthand experience on the impact of partners on research participation. As for the influence of family members, such as mothers and mothers-in-law, although they played down by a few pregnant and MINA women with the pretext that this *“is not something that concerns them”* G1_15, for many the opinion of the extended family mattered. KIs echoed similar views and further indicated that partners and families’ opinion mattered more among individuals from *“lower socioeconomic status, where before doing anything they need to ask their husbands, or mothers, or mothers-in-law”* G3_07 and among individuals living in rural areas where extended family rules prevail.

### Study characteristics

(i) Burden of the study:

In terms of burden of the study, the study participants described time commitment, logistical inconvenience, and type of data collected as factors influencing their decision to participate. Time commitment was perhaps the most pivotal factor in the decision to participate in studies, and according to some, the only barrier. In relation to cohort study participation, all three group participants pointed to long-term commitment as a major barrier. All three groups also agreed that as long as the study is convenient and does not take them out of their comfort zone, they would be willing to participate. Finding the balance between participation and family, work, and other life commitments also emerged as pivotal factors in the decision to participate. Logistical inconvenience -such as transportation, travelling to complete the requirements of the test, taking time-off from work, or at times unexpected circumstances- may lead to attrition. Moreover, the type of data collected was also a factor for some participants. A few MINA women indicated that collecting blood and body fluid specimen was not a problem, but somehow recollection of food consumed within the last 24 h bothered one participant.

(ii) Ethical considerations*:*

Participants thought that ethical considerations related to privacy, transparency, flexibility and sharing results would improve acceptance. The type of host institution also mattered. For instance, a research study based in a reputable institution is highly valued by some participants.

(iii) Incentives:

In terms of incentives, although opinions varied, many subjects were motivated to participate because they received incentives. For some KIs, incentives played a dual effect, depending on the socioeconomic status.

(iv) Researcher interpersonal skills and physicians’ endorsement to participate:

The interpersonal skills of the researcher encouraged many pregnant and MINA women to participate and to remain in the study. Moreover, the physicians’ endorsement to participate in studies seemed to motivate subjects to participate.

## The interaction between the factors influencing participation

**Fig. 2:**
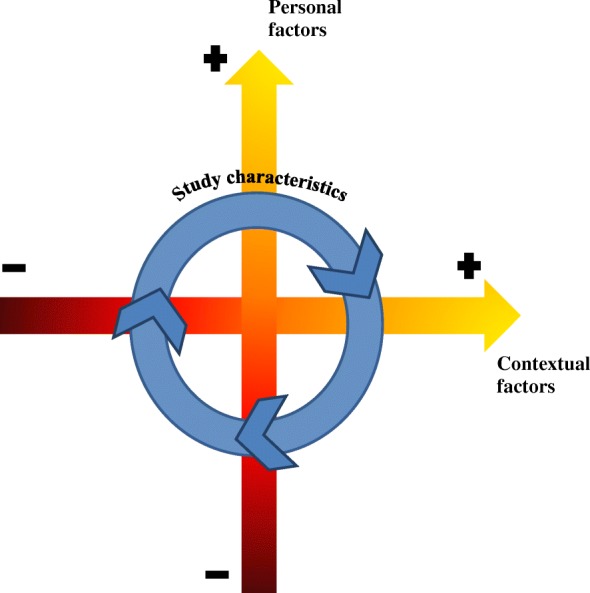
The interaction between the factors influencing participation in research studies

The thorough examination of the study results suggested an interaction among the three factors influencing participation including personal factors, environmental factors, and study characteristics. The quotes of the personal factors spanned a wide spectrum ranging from participants who are introverted, less confident, and not interested to those who are extroverted, confident, and interested in the general good. Similarly, contextual factors presented a range of answers from societies, families, and friends least to most supportive of subjects’ participation in research studies. Hence, these factors (personal and contextual) emerged as continuum rather than constant effectors of the decision to participate and were represented as y- and x- axes which intersect to form a dynamic four-quadrant interaction (Fig. 2). Individuals positioned on the lower-left quadrant were the least likely to participate; those on the upper-right quadrant were the most likely to participate; while those on the upper-left quadrant and the lower-right quadrant were indecisive, hence a good target for measures to improve participation. ‘Study characteristics’ seemed to affect the decision of pregnant women situated in any of the four quadrants; hence, this factor was shown in the graph as a dynamic effect encompassing the four quadrants.

## Recommendations to improve participation

Several recommendations were proposed by the MINA pregnant women and KIs to improve participation. These recommendations included enhancing exposure to research through the media (in all its forms), highlighting the successful stories from previous national and international research in improving human well-being. Furthermore, decreasing the burden of the study on participants was also identified. Since the physician’s involvement emerged as a key factor in affecting the decision to participate, decreasing the demand of the research on the physician’s time and commitment and enhancing communication were indicated as effective measures. Table 3 represented the specific recommendations, their respective quotes, and the factors that they correspond to as identified in this study.

**Table 3:** Specific recommendations, respective quotes, and factors they correspond to as identified in this study

Factors influencing participation	Recommendations	Quotes
Personal factors		
Previous understanding of the nature and purpose of research in general	Exposure of the target population to the importance of research by giving them examples of successful international studies that have resulted in positive change in health and well-being.	G1_17: “*If you can provide the moms with such information from studies that have been performed abroad, maybe then they will be more encouraged to participate. I think that people are most interested by the baby’s health, so they need something to confirm to them that what you are talking about really has an effect*.”
Contextual factors		
Exposure to research through the media	Enhancing the visibility of research in the social media, in the form of brochures, and on the institution’s website	G2_02:*“You could advertise the study on a Facebook page or you could write a small article about the study in a way to encourage women to participate. For example, I personally scroll through Facebook and access pages or articles that might be of interest to me or that might provide me with new information I am interested in.”*
Study characteristics		
Burden of the study	Going beyond academic institutions, where often the same patients receive frequent invitations to participate in research, by targeting community settings	G3_07:*“In our department there are a lot of ongoing studies and that’s why I think they become overwhelmed with so many study requests. Every patient who comes to deliver will be on 2 or 3 studies at least”*
	Spreading the data collection visits over a wider timeline	G1_12*:* *“It’s okay if it is just for 30 min every 2 weeks or every month its ok.”*
	Collecting data through email or via phone rather than meeting in person	G1_12:*“If it’s just a phone call she might take it when her baby is sleeping, but I imagine that leaving her baby at home and going somewhere for the sake of the study is not possible.”* G1_20: “*Do you have to do it in person, can’t you by email to fill a questionnaire”*
Incentives	Tailoring incentives to the population being studied	G3_12: *“If you go down to the Outpatient Department, where patients pay 5000 LL or 10 000LL to be seen and you tell them I will give you fifty dollars incentive, they will say yes I could use the fifty dollars. But if you come here to the private clinics and she is already paying 100$ to see me and you tell her you know what we are going to give an incentive of fifty dollars she will tell you I don’t care. You see what I mean?”*
Research interpersonal skills and physician endorsement to participate	Enhancing communication between principal investigators, research assistants and physicians	G3_01: *“I think that the people who are actually engaged in the study should really sit together and talk and discuss it a bit more. We didn’t really get the chance to convey our opinion about the study and what is actually happening. Maybe field workers don’t really have the experience that other people have, but we are on the field and we encounter the obstacles and the challenges. It is us who are in contact with the participants. So maybe we are a bit more aware of what’s wrong on field. Because on paper it is not as nice as on field; it doesn’t go as smoothly as it should. So I think more often we should have more meeting with the people involved in the study to simply just discuss things in more details and probably come up with better solutions to boost a little bit more the recruitment.”*
	Improving physician’s involvement in recruiting patients by flagging eligible patient’s record or sending frequent reminders about the study	G3_07: *“It would good if they [nurses] put a yellow mark in order to know that this is potential case for the study”* G3_10: *“Emails would help as a constant reminder, yes it’s good that physicians could approach the patients as a starting point, so to be reminded whether by email or phone calls at times we can maybe call the physicians and just remind them”*

## Discussion

This is the first qualitative study in the Middle East and North Africa (MENA) region to examine the factors that motivate/deter pregnant women from participating in research, and to explore subjects’ recommendations to improve participation.

In this study, and in accordance with previous reports, personal and contextual factors as well as study characteristics were found to affect participation. For instance, similar to our findings, in the Mayo Mammography Health Study (MMHS), altruism, as personal trait, was found to be a motivating factor for participation. In this study, authors suggested including, a personal altruistic note on the outside of the study invitation envelope, such as ‘Open this if you are interested in helping to prevent breast cancer’ helped increasing recruitment [37]. Furthermore, previous research also underscored the role of contextual factors in influencing the decision to participate, whereby the opinion of family members and friends was found to be a significant determinant of the decision to participate in breast cancer and other research [18, 19]. Most of the study characteristics that influenced participation in this study were repeatedly reported in the literature and included time commitment particularly in longitudinal studies [38, 39], survey overload [7, 37, 40, 41], and the need for additional visits [18], type of data collected [38, 42], ethical considerations including fear of breach in confidentiality and distrust or general skepticism about research [13, 38], and use of incentives [13, 43, 44]. The positive role of the physician on subjects’ recruitment found in this study seemed to be a common denominator to most investigations of factors affecting participation in research and could be explained by the fact that participants either felt reassured [12] or felt a sense of obligation to their healthcare provider [45, 46] and in some instances did not permit access to their records unless their treating physician was aware of this access [19].

In addition to highlighting the importance of contextual and personal factors and study characteristics in influencing the decision to participate in research, this study is the first to offer an understanding of the co-influence and interaction of the aforementioned factors. The proposed conceptual framework depicts a four quadrant chart pointing to an axial interaction between personal and contextual co-influenced by study characteristics. We postulate that those factors need to work together to reach the aspired level of participation among pregnant women. For instance, even if the subject presented with the utmost positive attitude towards research, a discouraging entourage (family and friends) could possibly present his/her participation. Furthermore, positive personality traits and a supporting context for participation in research may not be enough to support participation in research if the study presents significant logistical challenges (such as time commitment). Therefore, in order to improve participation in research, this study proposed a holistic framework integrating contextual,and personal factors as well as study characteristics. This framework also identifies targeted subjects for intervention aimed at increasing participation. The intersection of axes of personal and contextual factors each with a gradient from left-to-right and low-to-high resulted in four quadrants.

Specifically, two groups of subjects were identified as potential targets for recruitment (those at the upper-left and lower-right quadrants of Fig. 2): (1) those who have positive altruistic factors but yet have discouraging contextual factors and (2) those who, despite their supportive environment, remained less confident and not interested in participation. Tailoring interventions and recruitment strategies to motivate these two groups seemed to be promising to improve recruitment as described below. On the other hand, those on the lower-left are least amenable to change and could constitute a challenging target to interventions.

The proposed conceptual framework is meant to be used as an instrumental tool for researchers interested in improving recruitment not only in LMIC but also globally. For instance, this framework advocates that the role of health researchers is not only limited to manipulating the study characteristics, but also has to discuss and address the personal and contextual factors influencing their targeted population.

## Recommendations to improve participation in research studies

### Increasing visibility of research studies in the mainstream and social media

Poor public awareness of research was identified as a major barrier for research participation among different population groups [42, 45]. Pioneering examples of using mainstream media to raise awareness and as a tool to encourage the public to participate in clinical research include the March of Dimes campaign to raise support for clinical research on infantile paralysis and in the Love/Avon Army of Women to encourage women to participate in breast cancer trials [47].

Leveraging the power of social media in reaching the population is increasingly being recommended to increase recruitment [48–50]. The most popular social media platforms include Facebook, Twitter, Linkedin, Youtube, Timblir, and Instagram The empirical evidence on the impact of those various channels is only emerging. In a systematic review, Whitaker et al. examined the impact of Facebook on recruitment of participants for health research purposes and found that Facebook is a useful tool to increase recruitment [51]. Its benefits include reduced costs, shorter recruitment period, and increase in recruitment [51]. But many studies recommended also caution in using social media. Social media can potentially lead to inequitable recruitment favoring younger, more educated and more technology savvy individuals.

### Expanding and reaching out to different institutions and targeting interested population

Similar to our findings, Newington and Metcalfe suggested establishing integrated clinical and academic teams to increase the sampling frame of potential eligible participants and [42]. Furthermore, Treweek, [42, 52] recommended the Opt-in approach to specifically target those who are interested as an important strategy. Perhaps, our different academic institutions (in-patient and out-patient) can ask upon admission patients whether they would be interested in participating in research and the best channel to contact them, i.e. phone or email, hence creating a database for potential use by researchers.

### Interpersonal skills of recruiters and training the art of recruitment

Recruiters’ skills were perceived as important factors in improving recruitment [42]. Experienced recruiters who have developed overtime the art of recruitment are more effective in recruiting than the less experienced. The latter would benefit from a generic training on the art of recruitment before engaging in a research project. Further, Nishimura et al. (2013) [53] emphasized the need for frequent interaction between researchers and recruiters to discuss challenges and ways to improve recruitment are crucial [53], which was also suggested by KIs in our study. Finally, personality characteristics such as warmth, empathy, and assertiveness inspiring trust among potential participants will encourage participation [54].

### Maximize physician involvement in recruitment

In a culture like Lebanon where physicians’ opinions are highly valued, it is of utmost importance to increase physicians’ involvement in the recruitment process. Newington et al. (2014) referred to recruitment as a “team effort” [42]. Successful recruitment is better off when physicians mention the study to the participants before being approached by recruiters [42]. In the same vein, KIs in our study suggested strategies to increase physician’s involvement such as using flags as reminders to prompt them to discuss the research project with eligible participants.

### Incentives

Our results indicate a dual effect of incentives on participation. On one hand avoid burdening participants by providing compensation for their time and out- of pocket payment was perceived as a necessity, while others noted that incentives are futile and not needed. The semantic of this disagreement points to the importance of being considerate while planning for research. Researchers who opt to use incentives need to provide explanation for why an incentive is necessary for their study: Is it to avoid additional financial burden on individuals or is it to attract individuals to participate? If the main motive is to attract more individuals to participate, then ethical considerations may arise about potential financial motivation as risk factor in participation [55], while research considerations regarding disproportionally attracting individuals from lower socioeconomic status to participate may create a bias sample [56]. Similar to our findings, a systematic review, Stunkel et al. showed that financial reward can be a primary motivator for healthy volunteers, but non-financial motivation such as contributing to science, altruism, and personal interest are important motivators aside from financial [44]. It is perhaps recommended that the research team discuss the various motivators for participation before deciding on the form and amount of incentive being used if any.

### Strengths and limitations

This study is one of a few being conducted in the MENA region. It adds to previous work being done globally on research recruitment facilitators and challenges. We used the perspectives of potential, existing and KIs to elicit their understanding of the facilitators and barriers for participation in research. We developed a conceptual framework to synthesize those factors. However, further work is needed to refine this framework and to ascertain whether it can be translated into other geographical locations and population groups. The applicability of this framework is limited by the fact that different population groups, such as older, men and younger individuals, may have similar and also other perspectives on participation specific to their demographic characteristics. In addition, our research question mainly concerned healthy participants and not participants who suffer from diseases who have different factors motivating them to participate in research.

## Conclusion

We found three factors influencing participation in research: personal factors, contextual factors, and study characteristics. A thorough examination of these factors identified two groups of subjects who may be promising targets for interventions to enhance recruitment: subjects who have positive altruistic factors but yet have discouraging environmental factors and subjects, who, despite their supportive environment remained less confident and not much interested in participation. Several recommendations to improve recruitment were suggested including increasing visibility of research in the media, using social media for recruitment, increasing sampling frame, creating an opt-in database, pre-recruitment preparation, increasing physician’s involvement, and carefully considering incentives. Further research is needed to explore other population group perspectives and appropriate strategies to optimize participation.

## Additional files

Additional file 1: Topic guide. (DOCX 15 kb)

Additional file 2: Sociodemographic questionnaire for participants of group 1 and group 2. (DOCX 16 kb)

Additional file 3: Sociodemographic questionnaire for participants of group 3. (DOCX 14 kb)

Additional file 4: COnsolidated criteria for REporting Qualitative research. (PDF 602 kb)

## Abbreviations

COREQ: COnsolidated criteria for REporting Qualitative research
KIs: Key Informants
LMIC: Low and Middle Income Countries
MENA: Middle East and North Africa
MINA: Mother and Infant Nutrition Assessment
TDF: Theoretical Domains Framework
