# Supplementary material for: The interaction of personal, contextual, and study characteristics and their effect on recruitment and participation of pregnant women in research: a qualitative study in Lebanon
Source: BMC Med Res Methodol. 2018 Nov 29;18:155. doi: 10.1186/s12874-018-0616-5 (PMC6267028; doi:10.1186/s12874-018-0616-5)
Supplement: Supplementary file 1 — Topic guide. (DOCX 15 kb) [file 12874_2018_616_MOESM1_ESM.docx]

**Additional File 1: Topic Guide**

| **Topic guide for group 1** | | |
| --- | --- | --- |
| **Domain** | **Question** | |
| Knowledge about medical research | - Are you familiar with the concept of “medical research”? - Are you familiar with the concept of “cohort study”? | |
| Skills | - Have you ever participated in any research project? - Have you ever participated in a cohort study? | |
| Beliefs about capabilities of participation | - If you were to be invited to enroll in a cohort study, do you think you would accept to participate? | |
| Beliefs about consequences of participation | - What would be the advantages and disadvantages of committing to participate in a cohort study? | |
| Motivation to participate | - Walk me through the process that would guide your decision whether to participate or not in a cohort study | |
| Social influences | - Do you know any friends or family who participated in cohort studies? | |
| Characteristics of the research study | - In your opinion, what do you think can be improved in order increase participation in cohort studies? | |
| **Topic guide for group 2** | | |
| **Domain** | | **Question** |
| Knowledge about medical research | | - What do you know now about cohort study that you did not know before? |
| Skills | | - What skills are required to be able to participate in a cohort study? |
| Beliefs about capabilities of participation | | - Why did you decide to enroll in a cohort study? - What did you decide to remain in the cohort study? |
| Beliefs about consequences of participation | | - What are the advantages and disadvantages of committing to participate in a cohort study? |
| Motivation to participate | | - Walk me through the process that guided your decision to participate in a cohort study - Walk me through the process that guided your decision to remain in a cohort study |
| Social influences | | - Do you know any friends or family who participated in cohort studies? |
| Characteristics of the research study | | - In your opinion, what do you think can be improved in order improve enrollment and retention in cohort studies? |
| **Topic guide for group 3** | | |
| **Domain** | | **Question** |
| Skills | | - What skills are needed in order to recruit individuals to participate in a cohort study? |
| Beliefs about capabilities of participation | | - What were the facilitators you encountered while recruiting participants? - What were the barriers you encountered while recruiting participants? - Do you think you have the skills to recruit participants? - Did you have any concerns about how the recruitment process is going? |
| Beliefs about consequences of participation | | - What do you think the impact of participation in cohort studies will have on patients? |
| Motivation to participate | | - Walk me through the process that would guide you while recruiting participants to a cohort study |
| Social influences | | - To what extent you think that having colleagues or friends conducting research would influence your decision to participate in conducting a research (or we can be more specific by suggesting to recruit participants to a cohort study) |
| Characteristics of the research study | | - In your opinion, what do you think can be improved in order improve recruitment to cohort studies? |
