# Supplementary material for: The interaction of personal, contextual, and study characteristics and their effect on recruitment and participation of pregnant women in research: a qualitative study in Lebanon
Source: BMC Med Res Methodol. 2018 Nov 29;18:155. doi: 10.1186/s12874-018-0616-5 (PMC6267028; doi:10.1186/s12874-018-0616-5)
Supplement: Supplementary file 2 — Sociodemographic questionnaire for participants of group 1 and group 2. (DOCX 16 kb) [file 12874_2018_616_MOESM2_ESM.docx]

**Additional File 2: Sociodemographic questionnaire for participants of group 1 and group 2**

1. **What is your date of birth (DD/MM/YYYY)?** ____/_____/_______
2. **What is your nationality?**
3. **What is the highest educational level that you have achieved?**
   1. No schooling
   2. Primary school
   3. Intermediate school
   4. High school
   5. Technical diploma
   6. University degree
   7. Refused to answer
4. **Did you specialize in a health-related major (medicine, biology, public health, pharmacy, etc.)?**

**(Skip if woman did not receive a technical diploma or university degree)**

1. Yes
2. No
3. **What kind of work do you do?**
4. Housewife/homemaker
5. Employee, full-time, *please specify:* _____________________
6. Employee, part-time, *please specify:* _____________________
7. Self-employed, *please specify:* _____________________
8. **Which area of Lebanon do you live in?**
9. Beirut
10. Mount Lebanon
11. South
12. Nabatiyeh
13. North
14. Bekaa
15. **What is your husband’s age?**  ______ years
16. **What is the highest educational level that your husband has achieved?**
17. No schooling
18. Primary school
19. Intermediate school
20. High school
21. Technical diploma
22. University degree
23. Refused to answer
24. **What kind of work does your husband do?**
25. Not working
26. Not working, but looking for a job
27. Employee, full-time
28. Employee, part-time
29. Self-employed, please specify _________________
30. Other, *please specify*: ________________________
31. **What is the monthly income of the family (L.L.) (this includes the sum of salaries of the woman and her husband, income coming from relatives, and income coming from renting a house, land, or other assets)?**
32. Less than 600,000 (less than $ 400)
33. 600,001 – 999,999 ($401 – $666.9)
34. 1,000,000 – 1,499,000 ($ 667 – $999.9)
35. 1,500,000 – 1,999,000 ($ 1,000 - $1,332.9)
36. 2,000,000 – 2,499,000 ($ 1333 - $ 1,666.9)
37. 2,500,000 – 2,999,000 ($1,667 – $1,999.9)
38. Greater or equal to 3,000,000 (greater or equal to $ 2,000)
39. Don’t know/Not sure
40. Refused to answer
