# Supplementary material for: The interaction of personal, contextual, and study characteristics and their effect on recruitment and participation of pregnant women in research: a qualitative study in Lebanon
Source: BMC Med Res Methodol. 2018 Nov 29;18:155. doi: 10.1186/s12874-018-0616-5 (PMC6267028; doi:10.1186/s12874-018-0616-5)
Supplement: Supplementary file 3 — Sociodemographic questionnaire for participants of group 3. (DOCX 14 kb) [file 12874_2018_616_MOESM3_ESM.docx]

**Additional File 3: Sociodemographic questionnaire for participants of group 3**

1. **Gender:** Male / Female
2. **Date of birth (DD/MM/YYYY)?** ____/_____/_______
3. **Nationality:**
4. **Highest level of education:**
5. **Specialty/job title:**
6. **Years of experience:**
7. **Previous research experience that involved subjects recruitment (other than the MINA):**
   1. No
   2. Yes. *Please specify*: _______________________
8. **Average number of pregnant women contacted per day:**
